# Using Small-Area Analysis to Estimate County-Level Racial Disparities in Obesity Demonstrating the Necessity of Targeted Interventions

**DOI:** 10.3390/ijerph110100418

**Published:** 2013-12-27

**Authors:** Lucy D’Agostino-McGowan, Renee L. Gennarelli, Sarah A. Lyons, Melody S. Goodman

**Affiliations:** Division of Public Health Sciences, Department of Surgery, Washington University School of Medicine, St. Louis, MO 63110, USA; E-Mails: gennarellir@wudosis.wustl.edu (R.L.G.); lyonss@wudosis.wustl.edu (S.A.L.); goodmanm@wudosis.wustl.edu (M.S.G.)

**Keywords:** obesity, disparities, small-area analysis, multilevel regression, targeted interventions

## Abstract

Data on the national and state levels is often used to inform policy decisions and strategies designed to reduce racial disparities in obesity. Obesity-related health outcomes are realized on the individual level, and policies based on state and national-level data may be inappropriate due to the variations in health outcomes within and between states. To examine county-level variation of obesity within states, we use a small-area analysis technique to fill the void for county-level obesity data by race. Five years of Behavioral Risk Factor Surveillance System data are used to estimate the prevalence of obesity by county, both overall and race-stratified. A modified weighting system is used based on demographics at the county level using 2010 census data. We fit a multilevel reweighted regression model to obtain county-level prevalence estimates by race. We compare the distribution of prevalence estimates of non-Hispanic Blacks to non-Hispanic Whites. For 25 of the 26 states included in our analysis there is a statistically significant difference between within-state county-level average obesity prevalence rates for non-Hispanic Whites and non-Hispanic Blacks. This study provides information needed to target disparities interventions and resources to the local areas with greatest need; it also identifies the necessity of doing so.

## 1. Introduction

Obesity is known to drastically increase the risk of chronic diseases and is associated with excess morbidity and mortality [1]. The prevalence rates of obesity in the United States remain higher than in most developed countries [2,3]. In 2009–2010, over 35% of adults were considered obese [4]. The rate of obesity is greater than 25% in 41 states, and 13 of these states have rates over 30%. In fact, there is no state with a prevalence rate of obesity lower than 20% [2]. These statistics, however, do not fully capture the obesity epidemic. We hypothesize that state and nation-wide obesity prevalence rates mask the variability of outcomes across smaller geographic areas and the existence of racial disparities within these areas [5]. 

The policies and strategies to reduce racial health disparities are often implemented on the national and state levels, informed by national and state-level data. These levels are often too far removed from the individual level where health outcomes are realized; a shift in focus to the local level may be necessary to accelerate progress in reducing racial health disparities. The lack of lower-level data hinders the effective evaluation of public health policy, programs, and interventions that occur at the local level [6]. The cost-prohibitive nature of data collection on the local, zip code, or county level has led us to implement small-area analysis techniques on publicly available state-level data to obtain county-level estimates [7,8,9]. These estimates are more informative than larger-level estimates and will be useful in implementing efficient public health interventions.

Survey data from the Behavioral Risk Factor Surveillance System (BRFSS) is commonly used to estimate the prevalence of chronic disease. The BRFSS telephone survey collects data on preventative health practices and risk behaviors for chronic diseases in adults. The data is collected in a uniform manner across all states [10]. The sampling design and weighting scheme is structured so that BRFSS data can only be used to calculate valid direct estimates of prevalence at the state or higher geographic levels [10,11]. Reweighting BRFSS data allows for the calculation of obesity prevalence estimates at the county-level.

In 2010, when stratified by race, 37% of non-Hispanic Black adults in the United States were obese, as compared to 26% of non-Hispanic Whites [12]. We aim to hone in on this 11% absolute difference by examining obesity data by race on the county level. It is a common assumption that racial disparities calculated on the national and state level hold true on the county level. However, adequate race-stratified obesity data from most counties is not available to support or dispute this claim. In order to produce obesity prevalence estimates by race on the county level, we reweight BRFSS data and use a multilevel regression model [7,13,14]. These prevalence estimates are then utilized to estimate county-level racial disparities. By demonstrating the variability of obesity by race throughout the country, we demonstrate the necessity of targeted interventions.

## 2. Methods

We combine five years (2006–2010) of BRFSS survey data to obtain the prevalence estimates for obesity, using the following two survey questions: “About how much do you weigh without shoes?” and “About how tall are you without shoes?”. If necessary, we convert the reported weight to kilograms and the reported height to meters. Body mass index (BMI) is calculated as:

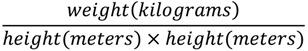
(1)

**Figure 1:**
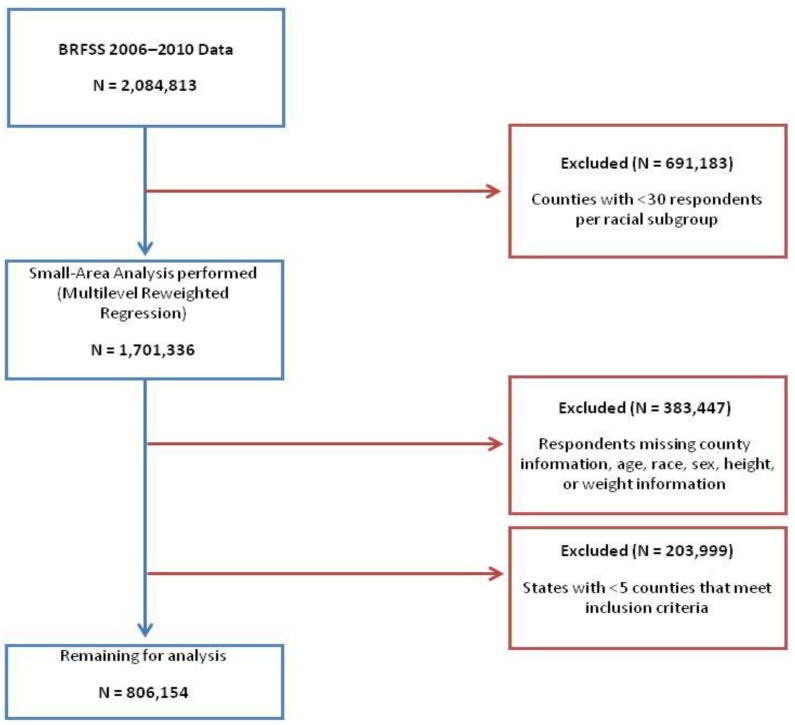
Study sample schema.

To determine obesity status, we used the National Institute of Health recommendation of a BMI greater than or equal to 30 as the cutoff [15]. Respondents with a calculated BMI greater than or equal to this cutoff are considered obese in this study. Those with missing information regarding age, sex, race, height, or weight were excluded from analysis. Counties with less than 30 respondents per racial subgroup were excluded from analysis. States with less than five counties that met our inclusion criteria were also excluded from our analysis (Figure 1).

### 2.1. Data Analysis

Analysis was completed using SAS/STAT version 9.3 (SAS Institute Inc., Cary, NC, USA). In a previous study, it was established that multilevel logistic regression models show the least amount of discrepancy when estimating county-level prevalence of chronic disease by race [7]. Here we use a similar model, incorporating modified county-level weights. In complex survey analyses, in order to make the survey data more representative of the general population, weights are assigned to each respondent. The values of these weights indicate how much each individual respondent will count in the statistical analyses to follow. BRFSS provides a weighting system that allows for direct estimates to be made on prevalence data at the state level. For the purposes of this study, a modified weighing system was used based on demographics (age, sex, and race) at the county level. This reweighting approach has been used previously to examine racial disparities in obesity in North Carolina [16]. Here, we applied multilevel reweighted regression to estimate the prevalence of obesity by race on the county level for all states that met our inclusion criteria. The equation for the multilevel reweighted regression is:

*logit*(*p_ij_*) = *X_ij_β* + *α_i_* (2)

In Equation (2), *X_ij_*= (*x_ij_*_1_, …*x_ijq_*) is the vector of q covariates, *β =* (*β*_1*,*_ … *β_q_*)*'* is the corresponding vector of fixed effects and *α_i_* is the random effect for county. The model includes demographic components: race, sex, and age group, as well as county-level data obtained from the 2010 census: County percent poverty, county urban (*versus* rural) status and proportion of adults older than 25 with less than a high school education (county education). County percent poverty is categorized as: counties with less than 15 percent poverty, counties with between 15 percent and less than 25 percent poverty, and counties with greater than or equal to 25 percent poverty. County urban status is categorized as counties that are less than or equal to 25 percent urban, counties that are between 25 and 75 percent urban, and counties that are greater than or equal to 75 percent urban. County Education is dichotomized as counties with less than 20 percent of adults older than 25 with less than a high school education and counties with greater than or equal to 20 percent of adults older than 25 with less than a high school education. The respondents are categorized by age in the following manner: ages 18 to 29, ages 30 to 49, ages 50 to 69, and ages greater than 70. We tested all possible interactions and included the following significant terms: sex and race, sex and age group, sex and county education, sex and county percent poverty, sex and county urban status, race and age group, race and county education, race and county percent poverty, race and county urban status, county education and county urban status, county education and county percent poverty, and county percent poverty and county urban status. 

After calculating the regression parameter estimates, we estimated the county-level prevalence rates by race. The county-level age-by-race-by-sex estimated prevalence is calculated from the regression model predictors, Equation (3):

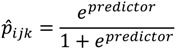
(3)

The county-level prevalence rates by race are then calculated with the following Formula (4):

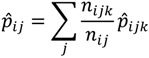
(4)

Here, 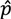
*_ij_* is the estimated prevalence of obesity in county *i* of race *j*; *n_ijk_*is the number of people in county *i* that are of race *j* and belong to age and sex demographic group *k*; *n_ij_* = ∑ *_j_ n_ijk_* is the total population in county *i* of race *j*; 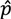
*_ijk_* is the estimated prevalence of obesity in county *i* for race *j* in demographic group *k.*

We compared the distribution of prevalence estimates of non-Hispanic Blacks to non-Hispanic Whites. After determining that the samples were normally distributed, we performed a two-sample test for proportions to compare the mean difference in estimated county-level prevalence of obesity and an F-test to examine the equality of the variances from the two distributions. We calculated the between-state variance in obesity prevalence using 2006–2010 BRFSS data. We calculated two variances, one using the 26 states included in this analysis and a second using all 50 states and the District of Columbia. We then compared the variance of obesity prevalence within-states to these between-state variance estimates and subsequently the race-stratified obesity prevalence estimates to the between-state estimates in order to examine the difference in variability within states as compared to between states. Here, for race-stratified variance we calculate the county-level prevalence estimate for the non-Hispanic White population and subsequently for the non-Hispanic Black population and include both estimates for each county within each state to produce a “race-stratified within-state” variance estimate.

## 3. Results and Discussion

After exclusion criteria, 806,154 individual respondents were included in the analysis (Table 1). 

**Table 1:** Study sample.

	N	Mean	Median	SD	Minimum	Maximum
**Observations**	806,154					
Observations per county		1,512.5	922.0	1,904.4	83.0	16,463.0
**Counties**	533					
Counties per state		20.5	12.0	17.0	5.0	57.0

Obesity prevalence rates were calculated by race (non-Hispanic-Black and non-Hispanic White) for each county. All 26 states had a higher mean prevalence rate of obesity for non-Hispanic Blacks than non-Hispanic Whites. A t-test was used to examine the racial differences in prevalence rates of obesity for states with normal distributions of county-level prevalence rates. Five of the 26 states had non-normal distributions of county-level prevalence rates. Wilcoxon signed-rank test was used in place of a t-test for these states. An F-test was conducted to compare variances; for all states included in the analysis, there is no evidence of unequal variances. For 25 of the 26 states, non-Hispanic Blacks had a statistically significantly higher mean prevalence of obesity as compared to non-Hispanic Whites (Table 2). Non-Hispanic Blacks in Colorado had a higher mean prevalence of obesity than non-Hispanic Whites, but it was not statistically significant (*p* = 0.0625). 

**Figure 2:**
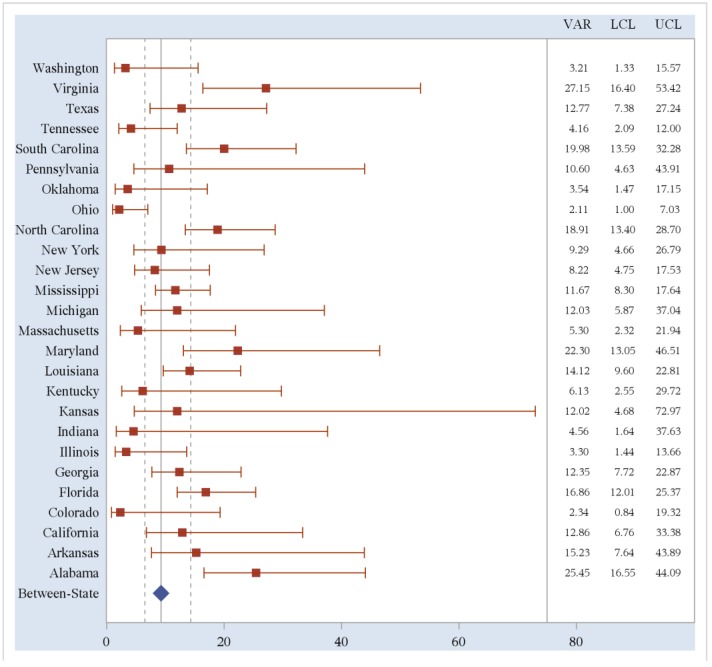
Within-State variability of obesity prevalence rates compared to between-state variability.

Within-state variances for obesity prevalence rates were then calculated using county-level estimates. We compared these variances to the between-state variance in obesity prevalence rates. States with less than five counties reporting data were excluded; 26 states (51%) are included in the analysis of within-state variability. We compared this within-state variability to both the between-state variance calculated using the 26 states included in this analysis and the variance calculated using all 50 states and the District of Columbia. Fifteen out of 26 states (58%) included in the analysis had a greater within-state variance estimates of obesity prevalence rates than the between-state variance for these 26 states (10.35, 95%CI 6.36–19.72). Sixteen out of 26 states (62%) included in the analysis had a greater within-state variability of obesity prevalence rates than the between-state variability for all 50 states and the District of Columbia (9.23, 95%CI: 6.49, 14.32). Two of these are statistically significantly different with non-overlapping confidence bounds. The within-state variability of obesity prevalence rates and 95% confidence limits are shown in Figure 2.

**Table 2:** Comparison of race-stratified prevalence means by state.

		White Prevalence of Obesity (%)		Black Prevalence of Obesity (%)		T-Test	
State	N	Mean	SD	Mean	SD	T Statistic	*p*-value
Alabama	34	24.46	4.62	31.60	4.83	6.23	<0.0001
Arkansas	12	23.17	3.90	29.22	3.72	3.89	0.0008
California	14	18.71	3.77	25.14	4.16	4.28	0.0002
Colorado	5	15.69	1.98	22.07	2.25	7.50 *	0.0625
Florida	57	21.22	3.87	27.58	4.73	7.86	<0.0001
Georgia	28	18.58	3.08	25.53	3.53	7.85	<0.0001
Illinois	8	18.17	1.38	24.61	2.37	6.63	<0.0001
Indiana	5	20.49	1.89	28.17	1.78	6.63	0.0002
Kansas	6	19.61	3.47	26.93	3.81	3.48	0.0059
Kentucky	7	22.00	2.89	29.73	2.38	14.00 *	0.0156
Louisiana	43	22.40	3.74	28.94	4.29	473.00 *	<0.0001
Maryland	21	21.46	4.33	28.09	4.63	115.50 *	<.0001
Massachusetts	8	17.43	2.46	24.42	2.68	5.42	<.0001
Michigan	11	21.56	3.33	29.35	3.46	5.38	<0.0001
Mississippi	56	23.57	3.13	29.77	3.38	10.06	<0.0001
New Jersey	20	18.57	2.97	25.60	2.72	7.82	<0.0001
New York	12	17.07	3.55	23.97	2.88	5.23	<0.0001
North Carolina	55	22.62	3.85	30.13	4.04	9.99	<0.0001
Ohio	10	21.54	2.16	29.61	2.26	8.17	<0.0001
Oklahoma	7	22.25	2.10	30.45	2.46	6.72	<0.0001
Pennsylvania	8	20.43	3.21	27.23	3.35	4.15	0.0010
South Carolina	43	22.95	3.75	30.37	3.94	8.94	<0.0001
Tennessee	12	21.45	2.43	28.23	1.84	7.70	<0.0001
Texas	20	20.25	3.68	27.48	4.06	5.90	<0.0001
Virginia	24	18.30	4.56	25.97	5.80	5.09	<0.0001
Washington	7	20.50	1.89	27.54	2.38	14.00 *	0.0156

Note: * Obesity Prevalence Rates had non-normal distributions. S-statistics were calculated in place of T-statistics using Wilcoxon signed-rank test.

The lower variability estimates of states, such as Ohio, indicate that the obesity prevalence rates for each county within the state are similar. In these cases, a state-wide intervention would be more appropriate than in a state such as Virginia where there is greater variability of obesity prevalence rates. Virginia, and other states with greater within-state variability, has a wide variety of obesity prevalence rates among counties. Therefore, local targeted interventions would be the most useful in reducing obesity in these states.

The forest plot above shows the variance point estimates and 95% confidence intervals for all 26 states included in the analysis. The red squares represent the variance estimates while the horizontal red lines represent the confidence intervals for each state variance estimate. The blue diamond represents the between-state variance for all 50 states and the District of Columbia while the vertical, gray dotted lines represent the 95% confidence interval for this between-state variance. To the right, the column labeled “VAR” provides the numeric variance estimates for each state while the columns labeled “LCL” and “UCL” represent the lower and upper confidence limits, respectively, for the variance estimates.

**Figure 3:**
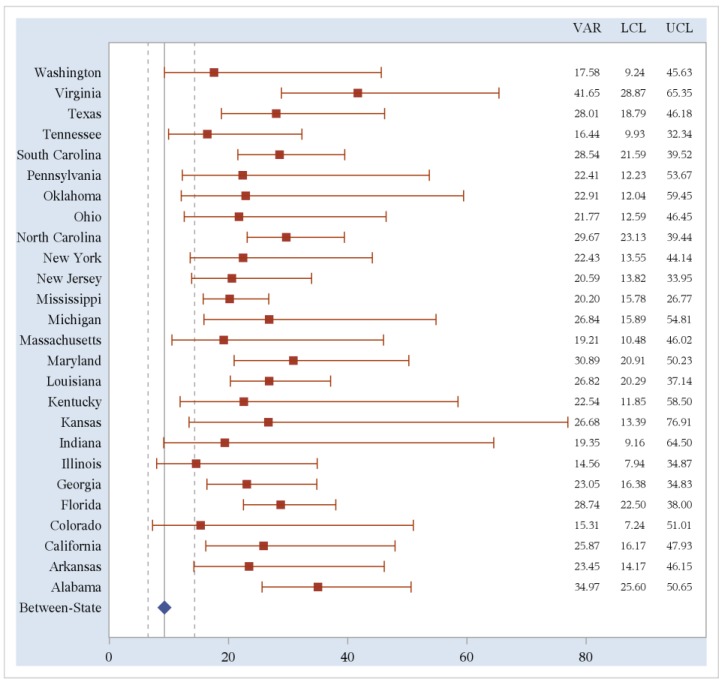
Race-stratified within-state variability of obesity prevalence rates.

Within-state variance was then calculated again for each state with race-specific prevalence rates included for each county. This race-stratified within-state variability aims to quantify the racial disparity among obesity rates in each state. The calculated race-stratified within-state variances were higher than both the un-stratified within-state variance and the overall between-state variance for all 26 states included in the analysis (Figure 3). This graph plots the variance of race-stratified county-level obesity prevalence by state along with 95% upper (UCL) and lower (LCL) confidence limits as compared to the variance of obesity prevalence of overall state-level estimates (Between-State). The variance estimates for all 26 states included in the analysis fall outside of the confidence bounds for the overall between-state variance, although the same cannot be said for the state-specific confidence bounds. In 12 (46%) of the 26 states, the variance estimate and its entire confidence bound fell completely outside of the overall between-state’s confidence bounds. Again, this highlights the variability within states and the necessity of tailored, culturally competent, and region-specific community based interventions to address the obesity problem in our nation. 

Since within-state variance distributions are normal for both races, a two sample t-test was used to compare mean variance between the two groups. The mean race-stratified within-state variance (24.22) is significantly higher than that of the un-stratified within-state variance (11.40) (*p* < 0.0001). The mean un-stratified within-state variance is not statistically significantly higher than the between-state variance of 9.27 (*p* = 0.1433), however when stratified by race, the within-state variance is statistically significantly higher than the between-state variance (*p* ≤ 0.0001).

## 4. Conclusions

Obesity prevalence rates on the state or national level are potentially misleading and overlook the information provided by a more local approach. Small-area analysis of publically available data is a cost-effective and useful way to produce local-level prevalence estimates. Over half of the states included in our analysis have greater variability in obesity prevalence between their counties than the overall variability in obesity prevalence between states. Perhaps states are not an appropriate subunit to examine obesity prevalence in the United States. Counties with high prevalence rates might be overlooked because they are in a state with an overall low prevalence. For example, Virginia has the largest amount of county-level variability and yet ranked 29th in overall obesity prevalence. By giving Virginia less attention due to its relatively low prevalence, Virginia counties with higher obesity prevalence may not receive the resources they need. When stratifying by race, the estimates are even more variable, further necessitating the use of race-stratified county-level data.

One limitation of this study is the nature of the available data. Due to the BRFSS sampling and the response rates by race, many counties had less than 30 non-Hispanic Black respondents, and therefore could not be included in our analysis. This potentially led to a misestimation of within-state variance. In order to control for this, we excluded states with less than 5 counties and included 95% confidence limits for the variance estimates. Another potential limitation is the assumption that self-report bias does not vary across data levels. A recent study has shown that there is a geographic pattern in self-reported height and weight, causing estimates and rankings to be misleading [17]. This does, however, further emphasize the importance of looking within-states, rather than at states as a whole, given the propensity of people to self-report differently dependent on their region. BRFSS provides Selected Metropolitan/Micropolitan Area Risk Trends (SMART) datasets that include county-level information, however due to the weighting scheme, these estimates cannot be race stratified, and therefore are not comparable to our method [18]. 

The rising prevalence of obesity has major fiscal implications. In 1999, obesity attributed to 9.4% of the nation’s total health care expenditures [19]. Annual medical spending continues to grow in correlation with obesity rates, with a potential cost up to $147 billion per year [20]. Thus, it is a public health priority to reduce rates of obesity and relieve the economy of preventable costs. This study’s findings should be considered when deciding how to re-appropriate funding for the prevention and treatment of obesity. Due to the large amount of variability in obesity prevalence within states, it would be more cost-effective to implement these reduction strategies at a lower geographic level, such as county. This work also has future important implications for analysis on smaller geographic levels (e.g., zip code, census tract, census block). While it is not the case for the data used in this analysis, the emergence of geocoded data has important implications for the future directions of this work, as it increases our ability to define, redefine, and estimate on smaller geographic levels.
